# xenoGI 3: using the DTLOR model to reconstruct the evolution of gene families in clades of microbes

**DOI:** 10.1186/s12859-023-05410-0

**Published:** 2023-07-21

**Authors:** Nuo Liu, Tonatiuh A. Gonzalez, Jacob Fischer, Chan Hong, Michelle Johnson, Ross Mawhorter, Fabrizia Mugnatto, Rachael Soh, Shifa Somji, Joseph S. Wirth, Ran Libeskind-Hadas, Eliot C. Bush

**Affiliations:** 1 Department of Biology, Harvey Mudd College, Claremont, CA USA; 2 Department of Computer Science, Harvey Mudd College, Claremont, CA USA; 3 Department of Computer Science and Engineering, University of California Santa Cruz, Santa Cruz, CA USA; 4 Department of Integrated Sciences, Claremont McKenna College, Claremont, CA USA; 5 Present Address: Division of Foodborne, Waterborne, and Environmental Diseases, Centers for Disease Control and Prevention, Atlanta, GA USA

**Keywords:** Reconciliation, Gene family, Horizontal transfer, Genomic island

## Abstract

To understand genome evolution in a group of microbes, we need to know the timing of events such as duplications, deletions and horizontal transfers. A common approach is to perform a gene-tree / species-tree reconciliation. While a number of software packages perform this type of analysis, none are geared toward a complete reconstruction for all families in an entire clade. Here we describe an update to the xenoGI software package which allows users to perform such an analysis using the newly developed DTLOR (duplication-transfer-loss-origin-rearrangement) reconciliation model starting from genome sequences as input.

## Backgound

Microbes occupy a vast number of ecological niches, and the analysis of whole genomes is an important strategy for understanding how this came to be.

One common approach is to focus on gene content in particular groups of species [1–10]. Analyses of this type focus on the presence or absence of genes, and tend to place less emphasis on detailed phylogenetic relationships, and thus make less use of phylogenetic trees.

We can contrast this with a more fine-grained approach which focuses on the detailed evolutionary events occurring in a particular phylogeny, and which is carried out at the scale of individual gene families. A common way to infer the history of duplications, deletions and horizontal transfers in a particular gene family is to use a gene-tree / species-tree reconciliation. This involves mapping the gene tree onto the corresponding species tree and identifying the set of evolutionary events that account for differences between the two [11]. In this process, a model specifies the set of evolutionary events we choose to consider. One model that has commonly been applied to microbial genomes is the duplication-transfer-loss (DTL) model [12]. This model allows for gene duplication events, gene deletion (loss) events, as well as horizontal transfer events. Because of the importance of the latter in microbial evolution, the DTL model has been widely used in that context [13–16]. It is also currently an active area of algorithm and software development [17–22].

To date, software packages using reconciliation approaches have mostly taken phylogenetic trees as input (or a multiple alignment in the case of GeneRax [23]). Here we describe the addition of a DTL based approach to the xenoGI software package. It’s inclusion in this package allows a user to start from genbank files and ultimately obtain a set of genomic events for every gene family in the input genomes.

One limitation of the basic DTL model is that it allows for only a single entry of the gene tree into the species tree. Under DTL, a gene family can enter into the species tree either as a core gene, or via a single horizontal transfer event from outside. However it is not possible for it to enter multiple times via multiple horizontal transfer events from outside. This is problematic because there are many situations where in reality multiple entries have occurred [24, 25]. To better handle such situations, we recently developed a variation of DTL called DTLOR (this stands for duplication-transfer-loss-origin-rearrangement) [26]. This variant adds two new events. An origin event represents the entry of the gene tree into the species tree from outside. The DTLOR model allows there to be multiple such events. It also tracks the syntenic position of genes and allows genes to move to different locations via rearrangement events. Because of these changes, DTLOR is especially well suited to reconstructing the evolution of gene families in clades of closely related microbes.

## Results

### Assessing performance using simulations

For the purposes of testing DTLOR we modified a previously used genome evolution simulator [24]. Such simulations provide ground truth, recording all the relevant events: duplication (D), horizontal transfer within the focal-clade (T), loss (L, also known as deletion), origin (O, xeno-horizontal transfer from outside the clade being studied) and rearrangement (R, movement of genes from one location in the genome to another).

**Table 1:** Precision, recall and F1 values calculated from running xenoGI on the output of genome evolution simulations

	Precision	Recall	F1
Family gene content	0.48 ± 0.02	0.99 ± 0.01	0.64 ± 0.02
Origin	0.47 ± 0.02	0.97 ± 0.01	0.63 ± 0.02
Duplication	0.63 ± 0.03	0.9 ± 0.02	0.74 ± 0.03
Horizontal transfer within tree	0.6 ± 0.02	0.31 ± 0.03	0.4 ± 0.02
Loss	0.79 ± 0.02	0.62 ± 0.02	0.69 ± 0.01
Rearrangement	0.1 ± 0.02	0.33 ± 0.06	0.15 ± 0.03

To assess xenoGI’s performance we looked at how well it reconstructed the history of individual genes. For example, consider a gene with a duplication event occurring on a particular species tree branch. If xenoGI also shows a duplication event in that gene’s history on the same branch, then we consider it to have accurately reconstructed the gene’s history (with respect to duplications). We ran 20 simulations on randomly generated 6-species trees, analyzed the output with xenoGI, and calculated precision, recall and F1 values for each of the DTLOR events. Those values are given in Table 1.

As the table shows, recall (also known as sensitivity) values are highest for origin and duplication events (0.97 and 0.90), indicating that xenoGI is recovering the majority of these events. Recall is also high for family gene content (0.99) indicating it is correctly identifying which genes are in which families. Recall values for gene loss are intermediate (0.62), and they are lower for rearrangement and horizontal transfer within the clade (in the low 30 s). Precision values are more similar across events being between 0.47 and 0.79 for most events, except for rearrangement (0.10). These values suggest that xenoGI is best at reconstructing origin, duplication and loss events, and is less effective with horizontal transfer and rearrangement. This can also be seen in the F1 values (which are the harmonic mean of precision and recall).

### Several examples from microbial species

These examples illustrate how workers interested in the evolution of particular groups of species can use xenoGI as an exploratory method to identify gene families where particular evolutionary events have occurred.

#### *Vibrio* species example

We first consider some examples in a data set consisting of five *Vibrio* species: *V. cholerae* N16961 (assembly GCA_000006745.1), *V. fluvialis* ATCC 33809 (GCA_001558415.2), *V. qinghaiensis* Q67 (GCA_002257545.1), *V. parahaemolyticus* ATCC 17802 (GCA_001558495.2), and *V. tapetis* CECT4600 (GCA_900233005.1). We can use this data set to illustrate some of the functionality of xenoGI v3.

**Fig. 1:**
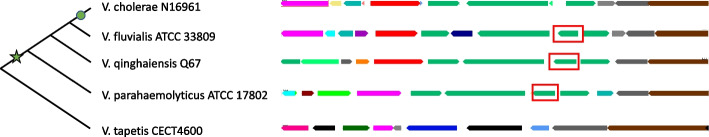
Illustration of a gene loss detected by xenoGI in a set of *Vibrio* species. The left side of the figure shows the phylogenetic tree for our five *Vibrio* strains. The right side shows one particular genomic region where a genomic island inserted. The island is present in *V. cholerae*, *V. fluvialis*, *V. qinghaiensis* and *V. parahaemolyticus*, having inserted on the lineage leading to *V. parahaemolyticus* (green star on the tree). The original insertion involved four genes, which are shown in green. However in *V. cholerae* only three are present. One gene, an AraC transcription factor, is present in *V. fluvialis*, *V. qinghaiensis* and *V. parahaemolyticus* (indicated by red boxes), but has been lost in *V. cholerae*. This gene was lost on the lineage leading to *V. cholerae* (green dot on the phylogenetic tree)

Figure 1 shows a phylogenetic tree for these strains, and one particular genomic region where xenoGI has identified an island consisting of 4 genes that inserted on the lineage leading to *V. parahaemolyticus* ATCC 17802. (Branch indicated by a green star on the tree). At the right of the figure, a genome browser representation of the region is shown for each species, with the inserted genes colored green. This region’s function is not known in detail, however it does contain a chitinase and a transcription factor in the AraC family.

Interestingly, xenoGI was able to recognize that the AraC transcription factor has been lost in *V. cholerae* N16961 (branch where loss/deletion occurred is indicated with a green dot). For the three species which possess this transcription factor gene, it is indicated with a red box in the genome browser representation. We identified this region by analyzing the xenoGI output for cases where an island inserted and one or more genes were subsequently lost.

**Fig. 2:**
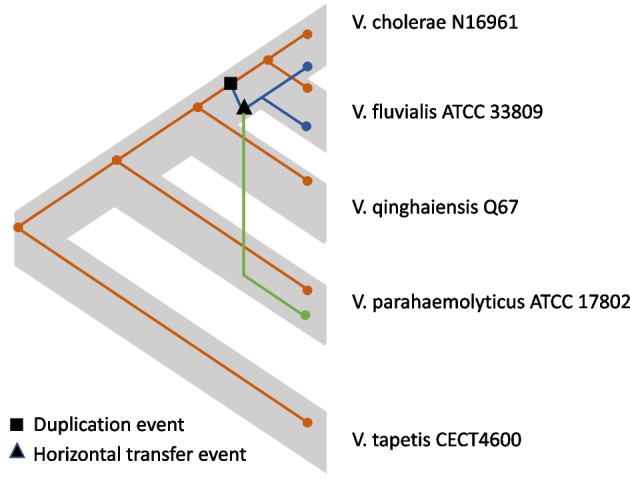
An example of a gene family with a duplication and a horizontal transfer event found by xenoGI in a set of *Vibrio* species. The species tree is shown with fat gray lines. The gene tree (thin orange, blue and green lines) for this family is mapped onto that species tree according to a reconciliation performed by xenoGI. The gene family is a core family which was present in the common ancestor. There is one copy of the gene present in each strain in the ancestral genomic location of this family. That is indicated by the orange gene tree. A duplication event occurred in the lineage leading to *V. cholerae* and *V. fluvialis*, after the divergence of *V. qinghaiensis* (black square). This duplication resulted in another copy of the gene in a different genomic location. This was inherited in *V. cholerae* and *V. fluvialis* (blue gene tree). This also became the source of a horizontal transfer to *V. parahaemolyticus* (black triangle and green line) which ended up in a third genomic location

Figure 2 shows another example from the same set of five *Vibrio* species. In this case it involves a single gene family where a duplication and a horizontal transfer event have occurred. The mdlB genes code for an ABC transporter protein whose function may be connected to fosfomycin resistance [27].

This is a core gene family in this group, present in the common ancestor of all five species in a single copy. That copy remains present in all species in the same location. The orange-colored gene tree in Fig. 2 represents this. The black box in the figure marks a duplication event that occurred in the lineage leading to *V. cholerae* and *V. fluvialis*, after the divergence of *V. qinghaiensis*. This duplication produced another copy in a different genomic location which was inherited in *V. cholerae* and *V. fluvialis* (blue gene tree). Before this copy diverged into those two species, it became the source of a horizontal transfer to *V. parahaemolyticus* (black triangle and green line).

#### Enteric species example

**Fig. 3:**
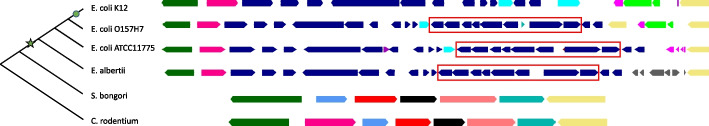
Evolution of the acid fitness island (AFI) and its genomic environs in a clade of enteric bacteria. At the left is a phylogenetic tree including six enteric species. At the right are displayed the genomic region containing the AFI, or the syntenic region in species that lack it. The displayed region extends from the treF (green) to gor (tan) genes. The AFI is shown in blue in the four species where it occurs, with the leftmost gene being gadA and the rightmost slp. The AFI inserted before the divergence of *E. albertii*, indicated on the tree by a green star. Subsequently, a subset of eight genes that were part of this original island (known as the heme transport locus and highlighted in the red boxes) were deleted in the lineage leading to *E. coli* K12, indicated on the tree by a green dot. xenoGI correctly identified the loss (deletion) event in each of these gene families

We next consider some examples in a data set consisting of six enteric bacteria: *C. rodentium* (Assembly GCF_000027085.1), *S. bongori* (GCA_000252995.1), *E. albertii* (GCA_001549955.1) and three strains of *E. coli*: *E. coli * ATCC11775 (GCA_003697165.2), *E. coli* O157H7 (GCA_000008865.1), and *E. coli* K12 (GCA_000005845.2). Figure 3 shows a phylogenetic tree for these strains, and genes in one particular genomic region which we will discuss next.

The acid fitness island (AFI) encodes a set of genes involved in resistance to acid stress including a glutamate decarboxylase enzyme [28]. This island is known to have originated on the lineage leading to the *E. coli*-*E. albertii* clade [24]. In our example enteric data set, xenoGI correctly places the origin of this island. In Fig. 3 on the left, the correct branch on the phylogenetic tree is marked with a green star. On the right the genes of the AFI island are indicated in dark blue for the *E. coli* and *E. albertii* strains. As noted by Bush et al. 2018, among these there is a set of 8 genes that was part of this original insertion, but which appears to have been deleted in the lineage leading to *E. coli* K12. These genes are involved in the scavenging of iron from hosts and have been referred to as the heme transport locus [29]. In Fig. 3, the red boxes in the genes for *E. albertii*, *E. coli * ATCC11775, and *E. coli* O157H7 indicate the position of the heme transport locus. Using DTLOR based reconciliations, xenoGI correctly identifies a loss event (i.e. a deletion) for these genes in the lineage leading to *E. coli* K12 (Fig. 3).

**Fig. 4:**
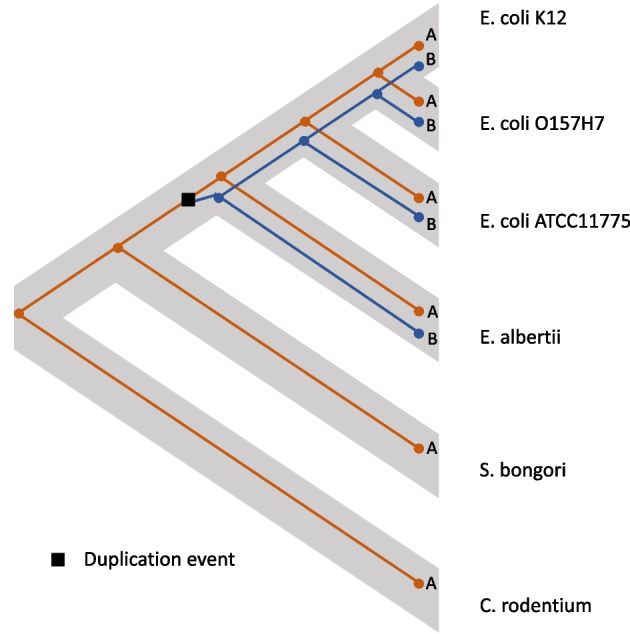
An illustration of the reconciliation of the gene tree for the pit genes against the species tree for a set of enteric bacteria, based on xenoGI output. The species tree is in the background in gray. The gene tree is drawn in the foreground, with the part corresponding to pitA genes in orange, the part corresponding to pitB genes in blue and the gene duplication event as a black square. Note that the gene duplication occurred after the divergence of *S. bongori*, but before the divergence of *E. albertii* from the *E. coli* clade

A gene family situated near to the AFI provides an example of a duplication event detected by DTLOR. Genes in this family are involved in phosphate transport [30]. One gene in the family, known as pitA is located about a dozen genes away from the AFI. In the *E. coli*-*E. albertii* clade there is also another paralog, pitB, which is located in another region of the genome. It has been suspected for some time that these two paralogues arose by a recent gene duplication [31]. The reconciliation output from xenoGI suggests this duplication happened in the lineage leading to the *E. coli*-*E. albertii* clade. Figure 4 is an illustration of this reconciliation.

**Fig. 5:**
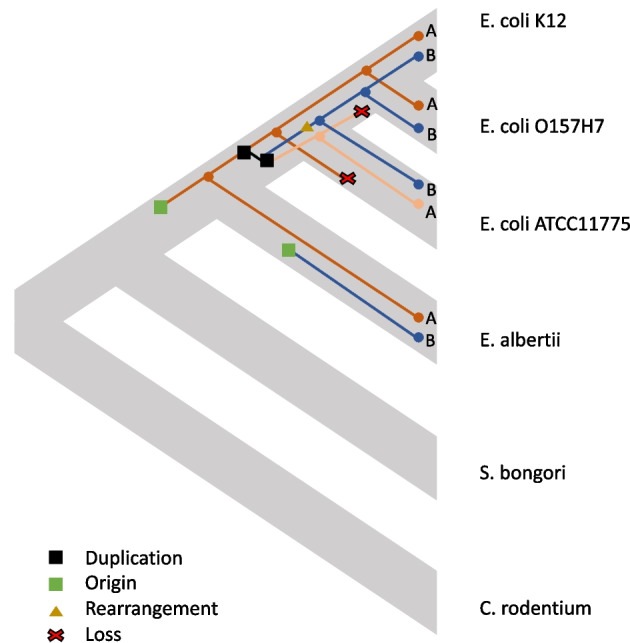
An illustration of the reconciliation of the gene tree for the gadA /gadB gene family against the species tree for a set of enteric bacteria, based on xenoGI output. The species tree is in the background in gray. The gene tree is drawn in the foreground, with the part corresponding to gadA genes in orange, the part corresponding to gadB genes in blue. This scenario involves several origin events, duplications and losses as well as a rearrangement event

An AFI gene of special interest is the glutamate carboxylase gene, gadA. Our reconciliation results for this gene family (which also includes gadB) involve several duplications, losses and rearrangements (Fig. 5). As we will discuss below, it is hard to be confident that this relatively complicated history represents what actually happened. However, it is a useful example to illustrate some of the limitations of the present method.

#### Comparison with DTL reconstructions from RANGER-DTL and Generax

We also compared xenoGI’s reconciliation results for these bacterial examples (Figs. 1, 2, 3, 4 and 5) with several existing software packages: RANGER-DTL v2 and Generax v2.0.4.

RANGER-DTL is a reconciliation package that takes a gene tree and a species tree as input. We tested it with the examples shown in Figs. 1, 2, 3, 4 and 5. In each of these five cases, RANGER-DTL came up with the same reconstruction as xenoGI. This is not particularly surprising because both use variants of the DTL algorithm.

Generax is a package which builds species-tree-aware gene trees. To do this, it makes use of a gene tree species tree reconciliation. It takes an alignment and a species tree as input. For the examples shown in Figs. 1, 3 and 4, Generax produces the same reconciliation that xenoGI does. However, for the examples shown in Figs. 2 and 5, the reconciliation is not the same. In both cases, Generax’s reconciliation involves a horizontal transfer event that goes from one branch to an ancestral branch. For example, it’s solution for the case shown in Fig. 5 involves a horizontal transfer event going from the *E. coli * ATCC11775 branch back to its own ancestor. Needless to say, such a solution is not realistic. The fact that Generax comes up with it has to do with the exact rules of the DTL reconciliation it is doing. xenoGI (and RANGER-DTL) don’t permit that sort of operation.

### Using xlMode to examine larger numbers of strains

Because of the scaling of run time and RAM usage with the number of genomes, it becomes difficult to use xenoGI much above 100 genomes. For this reason, we developed an xlMode which is able to run on larger numbers of strains. To test this we obtained a larger data set including all complete *Escherichia* genomes with a release date after Dec 21, 2020 as listed on NCBI. This consisted of 902 genomes. We ran xlMode with an initial random tree of 15 strains, and a final tree size of 20 strains. This completed in 3.5 days on our machine, using 50 cores.

The entire data set consisted of 4,236,584 genes in those 902 strains. Of these, 96.8% could be mapped onto a gene in the 20 strain scaffold tree. In this way, the xenoGI analysis of the scaffold tree provides a broad-brush summary of genome evolution in the larger data set.

## Discussion

The simulation results suggest that the DTLOR version of xenoGI is comparable to previous versions of the package in terms of the accuracy of its family delineation process. And that it’s able to identify various evolutionary events within families with relatively high accuracy.

Simulations also suggest that xenoGI-DTLOR is especially effective at reconstructing duplications and losses. While it is capable of reconstructing transfer and rearrangement events, it does so with lower accuracy.

As we noted above, the gadA/B case reveals some limitations of this kind of inference. The gadA/B reconciliation involves a number of duplications, losses and a rearrangement. Among other objections, that rearrangement (occurring on the branch leading to the *E. coli* clade) must move the gadB gene into the same syntenic location that gadB happened to insert on in the *E. albertii* branch. In such cases it is possible to experiment with different sets of DTLOR costs (and the package includes a script for doing this on single families). However, in this case, different DTLOR costs do not yield a simple and plausible explanation for evolution in this family. There are several reasons for this. First, in this case the sequence data underlying the gene tree does not allow for a robust and reliable gene tree. Bootstrap results with this multiple alignment included a number of poorly supported nodes. xenoGI uses GeneRax to calculate species tree aware gene trees, and this approach can help in terms of making more robust gene trees. However, if the underlying sequence data is inadequate, then we still end up with a gene tree that likely does not reflect reality. A second reason that reconstruction is challenging in the case of gadA is that this family may have experienced evolutionary events that are not included in the DTLOR model. In particular, it is thought that gadA and gadB have undergone gene conversion [32]. DTLOR includes transfer events where a gene is added in a particular syntenic location. Gene conversion across species can be approximated by a combination of an L and a T event. However, there is no mechanism for gene conversion from one location in the genome to another location in the same genome.

A goal for the future would be to include such events in the model. This could potentially lead to improvements. The challenge will be to find ways to do this without slowing the algorithm excessively.

## Conclusions

Our simulation results suggest that the DTLOR model can be used to successfully reconstruct the evolution of gene families in clades of closely related microbes. At present the accuracy is better for certain events (origin, duplication) than it is for others (rearrangement, horizontal transfer within the clade).

## Methods

### Overview

xenoGI is a command line program written in Python. It can be installed via pypi (https://pypi.org/project/xenoGI/), or by cloning the GitHub repository (https://github.com/ecbush/xenoGI). It can also be run inside a docker image (https://hub.docker.com/r/ecbush/xenogi).

Input consists of a set of GenBank (gbff) files and a species tree. The package also provides methods to aid in determining the species tree if that is not already known.

xenoGI follows three basic steps [24]. It first calculates a set of scores (similarity and synteny) between genes in the input genomes based on their protein sequences. It next creates gene families. Finally, it groups these families into islands with a common origin.

The initial steps of the analysis are similar to previous versions of xenoGI [24]. Briefly, they are as follows. The analysis begins with an all vs. all protein blast. Genes showing significant similarity must be above user-specified thresholds in terms of e-value, alignment coverage and percent identity. For those pairs of genes that do show significant similarity, we calculate a more refined raw score based on the global alignment of the proteins. We then calculate a set of synteny scores based on this (we no longer use the norm scores from previous versions). As a part of calculating synteny scores, we determine a hard-core gene set using the all-around best reciprocal hit method [24].

### Using reconciliation to build gene families

In order to reconstruct the evolutionary history within gene families, we have made changes to the family formation step. Previous versions of xenoGI did not make use of gene trees. We now build gene trees and reconcile them with the species tree using the DTLOR model in order to recover the various evolutionary events that have happened in their history.

#### Creating initial families and gene trees

Under the new approach, the first step is to create a large family of genes. This makes use of the all vs. all protein blast run in the previous step. We filter this output based on e-value, alignment coverage and percent identity. We then use single linkage clustering to construct an intial set of “blast families”.

These blast families can sometimes be very large making subsequent tree reconstruction steps unacceptably slow. We therefore break the biggest of them up into more manageable sizes. We do this by re-clustering with a higher score threshold for homology.

We next create gene trees for each cluster. We first create protein alignments using MUSCLE v5 [33]. Subsequent steps can either be based on these, or based on back aligned coding DNA made from them. We next use GeneRax to build species-tree aware gene trees based on these alignments [23].

Once the gene trees have been created, we again split the largest in order to improve efficiency in subsequent steps (this time principally for the reconciliation step). We identify gene trees with too many tips, and split on long branches, with an additional preference for branches that are internal (have a relatively balanced number of leaves on each side). The resulting families we refer to as *initial families*. Each initial family consists of a set of genes, and also has an associated unrooted gene tree.

#### Syntenic regions and locus families

The next step is to divide these initial families by syntenic region. To do this, we determine a set of thresholds for the core and regular synteny scores. We do this by examining the distribution of these scores for the set of hard-core gene families. Using the thresholds, we then divide the initial family into separate syntenic regions as follows. First, we create a graph containing each gene in the initial family as a node. An edge exists between two nodes (genes) when both the core and regular synteny scores are above threshold. We then divide this graph into connected components using single linkage clustering. Each connected component represents a set of genes in the same syntenic region. We refer to this as a locus family. An initial family will consist of one or more locus families.

#### Reconciliation using DTLOR

We next reconcile the gene tree against the species tree using the DTLOR (duplication-transfer-loss-origin-rearrangement) algorithm [34]. DTLOR is an extension for the DTL reconciliation model that allows multiple entry events into the species tree. To facilitate the recognition of such entry events, the model keeps track of the syntenic region of each gene as it evolves in the species tree. For a particular initial family, each locus family corresponds to a different syntenic region. The algorithm then reconstructs the evolution of the gene tree on the species tree. In addition to duplication, transfer, and loss events, the DTLOR model adds origin (O) events to indicate that a gene is transferred from outside of the species tree and rearrangement (R) events that account for changes in the syntenic regions of genes within the same the genome.

DTLOR reconciles a rooted gene tree onto a rooted species tree. The rooted species tree is either provided by the user or can be obtained using some functions in the package (see below). We use the DTLOR model to calculate a root for each gene tree by obtaining all possible rootings for each gene tree, and then reconcile each of these against the species tree. (Note that DTLOR, like DTL does not consider branch lengths, but only topology). We then keep the reconciliation and corresponding rooted gene tree with the lowest cost.

Having obtained a reconciliation for each initial family, we then use these to break up the initial families and their gene trees according to origin events. We call the new families that result from this *origin families*. Each origin family has a rooted gene tree associated with it. At the base of this tree is an origin event. Such origin events can either correspond to core genes (if they occur at the root of the species tree) or to horizontal transfer events (if they occur subsequent to the root).

When we create origin families from initial families, we also keep track of syntenic region information in the form of locus families. Like an initial family, an origin family will consist of one or more locus families. If there is only one locus family, then it will originate at the base of the origin family’s gene tree. If there is more than one, then somewhere in the gene tree there will be a rearrangement (R) event that led to another locus family.

### Locus islands, refinement, output

The next step is to group locus families that share a common origin into *locus islands*. This is done using a greedy approach described previously [24].

Sometimes there are multiple most parsimonious reconciliations (MPRs) for the gene tree corresponding to a particular initial family. To choose among these we use information from neighboring families. The logic behind this is the following. A particular MPR implies a particular partitioning of an initial family into origin and locus families. Because (for example) horizontal transfer events often bring in multiple genes in a single event, we have an expectation that neighboring genes on the chromosome will belong to origin and locus families that began on the same branch of the tree. Clearly this is not always so, but it often is, and it provides a basis to distinguish between equally parsimonious MPRs.

We call this the refinement step. After identifying reconciliations with multiple MPRs, we construct a test data set of nearby locus families. We then re-run locus island formation with each of the possible MPRs and find the MPR that yields the smallest number of locus islands. The solution represented by such an MPR is more consistent with nearby locus families, and thus more likely to be correct. After choosing the best MPR in such cases, we then re-run origin family and locus island formation for the whole data set.

As output, the package produces a set of text files which include a listing of all the locus islands, as well as a gene-by-gene representation of each genome. The origin of each gene, whether core or xeno horizontal transfer (that is horizontal transfer from outside the clade) is indicated. There is also a gene history string which gives the history of a gene from its origin until the tip of the gene tree. This consists of operations in the reconciliation model such as duplication, transfer (within the species tree), rearrangement etc. In addition to this text file output, it is also possible to produce bed files for visualization in a genome browser.

There are also interactive analysis functions in the package. Some of these will print out a representation of a particular locus island. Others give a detailed summary of origin and initial families, and the DTLOR-based reconciliation.

### Additional features

#### Capturing elements that repeatedly insert in the same location

Certain genomic elements are known to repeatedly insert in the same location, or to show very limited variation in terms of where they insert. An example of this is the SCCmec element in *Staphylococcus aureus* [35].

Such elements pose a challenge for our approach. Under typical DTLOR scoring parameters when independent insertion instances appear in the same syntenic region, they will be taken to result from a single insertion event in the common ancestor, followed by a number of losses.

To deal with this problem, we developed the following approach. If the user knows or suspects that certain elements repeatedly insert in the same location, then it is possible to identify them, and to have xenoGI use a different set of DTLOR parameters in this case. These alternate parameters are permissive to origin events. As a result, most insertions are labelled as independent events.

In this case, the user provides a file of genes belonging to said element. The system will then use a different set of DTLOR parameters for any family containing one or more of these genes. In particular, in this case the system uses a very large cost for D, T, L and R events, and a very small cost for O events.

#### Obtaining the species tree if it is not already known

The package includes functions to help with obtaining a species tree. This is typically run after score calculation and before family formation.

The first step is to create gene trees based on the hard-core gene families constructed during score calculation. MUSCLE is then used to create protein alignments for these [36]. We also optionally back-align this to DNA. From the alignments, FastTree is used to make gene trees [37]. And then ASTRAL is used to consolidate gene trees into a species tree [38].

#### Analyzing large numbers of strains

The approach described above is not practical to apply to data sets larger than 100 or so strains. To get around this we have developed a modified version that can be applied to data sets with many hundreds of strains. We do this by creating a representative species tree, containing only a subset of the strains. We then run xenoGI on this representative tree, obtaining family and locus island information for this smaller subset of strains. Finally, we map the genes from the entire data set back onto the families created from the representative subset. The runXlMode.py script which implements this is distributed in the *misc* directory of the GitHub repository.

There are four main steps to this method. First, we create a phylogenetic tree for the entire set of input genomes. The basic strategy is to identify a hard-core gene set and reconstruct the species tree based on those genes. To do this we use a modified version of the approach described above. As the number of genomes becomes large, the all vs. all blast used to identify the hard-core becomes limiting. To get around this problem we take a smaller random sample of genomes, and identify the hard-core gene set for this sample. Next, we blast families (several genes from each family) in this smaller hard-core against the remaining genomes. We then take the best hit from each of the remaining genomes eliminating families where there are no hits in one or more strains. After that we build gene trees for this hard-core data set, and reconstruct the species tree from it as described above.

The next major step is to distill the species tree down to a representative subset of strains. The goal is to create a distilled tree that contains as much diversity as possible from the full-sized tree. To do this we have implemented a version of the Treemmer algorithm which picks strains so as to maximize branch length [39]. It is also possible for the user to specify some (or all) of the strains that should be included in the representative subset.

Having created this distilled or representative tree, we run xenoGI on it and the corresponding genomes. From this we obtain a set of families and create a representative set of genes from these families.

We next blast all genes against this representative set, identifying unmapped genes (those with no blast similarity to any genes in our families). We next blast these unmapped genes against themselves and identify additional strains that we can include which will minimize the number of unmapped genes. We then construct a new scaffold tree including these strains, and run xenoGI on this new larger subset of strains.

Finally, we blast every remaining genome against the subset from the new, larger scaffold tree. Based on these blast results, we place genes from the remaining genomes into families from the subset as much as possible.

The final output of this method is a scaffold tree containing a subset of genomes, a statistic stating what percentage of the genes have been successfully mapped into families in the representative subset, and files containing that mapping.

## Data Availability

xenoGI is written in Python and released under the GPL v3.0 license. Its source code can be downloaded from github (https://github.com/ecbush/xenoGI). It can be run on any platform inside a docker image (https://hub.docker.com/r/ecbush/xenogi). It can also be installed and run directly on Linux (via pypi https://pypi.org/project/xenoGI/ or by downloading the repository from Github). In the latter case, users need to ensure the presence of NCBI blast+, MUSCLE v5, FastTree, GeneRax, and Python3 including the Biopython, Parasail, Scipy and Numpy packages.
